# Characterization of N- and C-terminal endogenously tagged Tyrosyl-DNA phosphodiesterase 2 (TDPT-1) *C. elegans* strains

**DOI:** 10.17912/micropub.biology.000540

**Published:** 2022-03-17

**Authors:** Nirajan Bhandari, Shannon C. Pfeiffer, Aimee Jaramillo-Lambert

**Affiliations:** 1 University of Delaware

## Abstract

We have generated Tyrosyl-DNA phosphodiesterase 2 (TDPT-1) *C. elegans* strains where CRISPR/Cas9 was used to endogenously tag the protein at either the C- or N-terminus and validated the functionality of the resulting tagged TDPT-1 proteins. We have found that both the N-terminally tagged ( *wrmScarlet::tdpt-1)* and C-terminally tagged ( *tdpt-1::3xflag* ) worm TDPT-1 does not affect embryonic viability compared to wild type. Using the N-terminally tagged *wrmScarlet::tdpt-1* strain we show, for the first time, that TDPT-1 is expressed in nuclei of the germ line and the soma. Moreover, we validate the expression of TDPT-1 at the protein level using the C-terminally tagged ( *tdpt-1::3xflag* ) strain.

**Figure 1. Characterization of N- and C-terminal endogenously tagged TDPT-1 strains:**
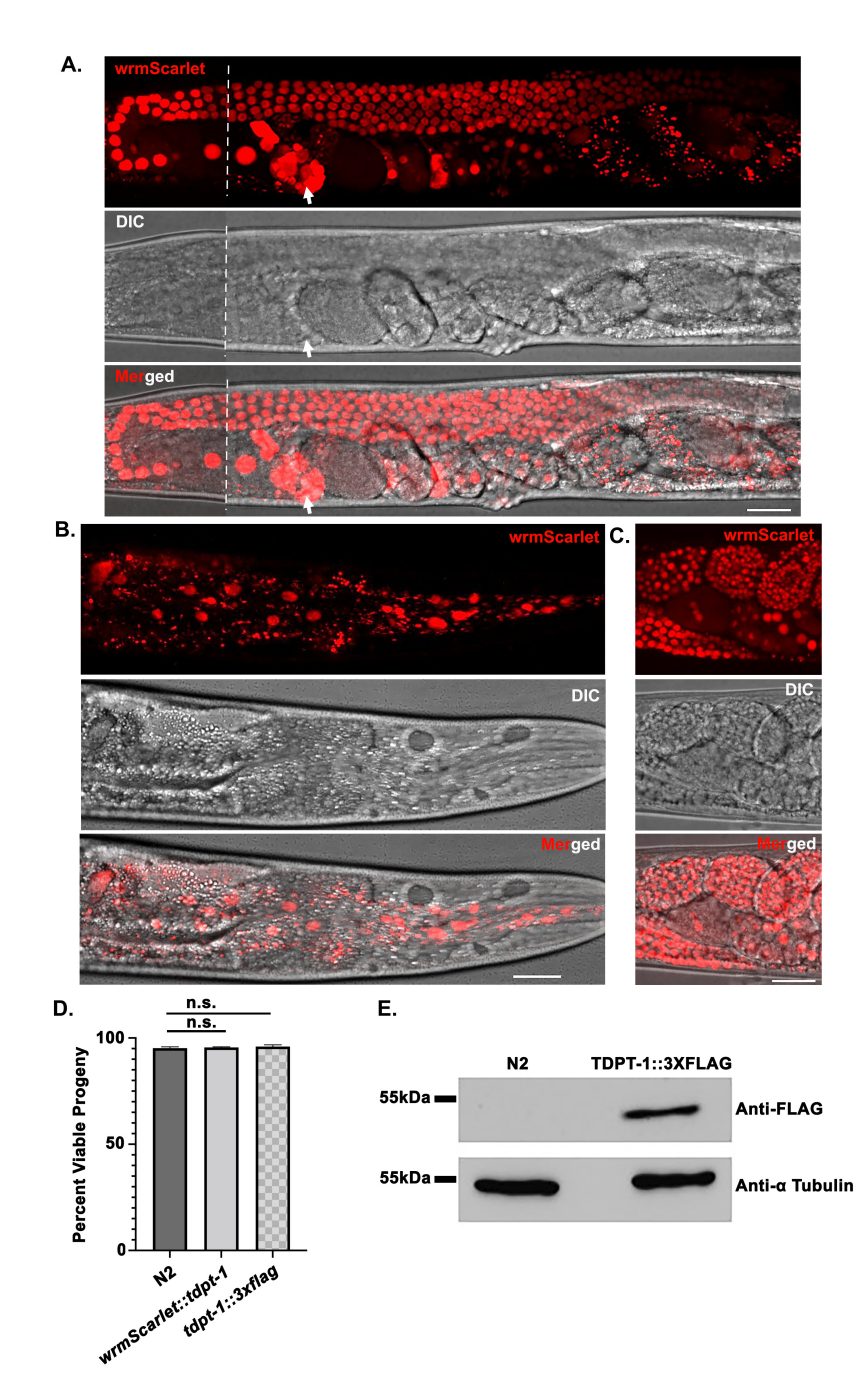
(A-B) Live confocal imaging of the *wrmScarlet::tdpt-1* strain. Top panels show wrmScarlet::TDPT-1 alone, the middle panels show the DIC image alone, and the lower panels show the merged images captured at 40X. Scale bars represent 25 μm. (A) Localization of wrmScarlet::TDPT-1 in the germ line of the hermaphrodite gonad. The white dashed line indicates a break/boundary of two images from the same worm that do not align perfectly. The spermatheca is indicated by white arrows. (B) Localization of wrmScarlet::TDPT-1 in somatic cells of the worm head. (C) Localization of wrmScarlet::TDPT-1 in embryos. (D) Percent viable progeny of tagged TDPT-1 strains compared to wild type (N2). The progeny of at least 30 hermaphrodites were scored for each genotype. The total number of progeny scored for each genotype was as follows: N2= 6,779; *wrmScarlet::tdpt-1* = 8,204; *tdpt-1::3xflag* = 7,149. Statistical analysis was performed using Student’s t-test, n.s. indicates not significant. (E) Western blot of TDPT-1 protein levels in wild type (N2) versus TDPT-1::3xFLAG *.* The TDPT-1::3XFLAG band (~44 kDa) is observed in lysates from the *tdpt-1::3xflag* strain and absent in wild type (N2) lysates. α Tubulin (50 kDa) control bands are present in both strains.

## Description

Tyrosyl-DNA phosphodiesterase 2 (TDP2) is a multifunctional protein involved in signal transduction, transcription regulation, and DNA repair (Esguerra et al., 2007; Pei et al., 2003; Pommier et al., 2016; Pype et al., 2000). In its role as a DNA repair enzyme, the phosphodiesterase activity of TDP2 is involved in the removal of trapped Top-2-DNA protein complexes (TOP-2-DPCs) (Nitiss, 2009; Schellenberg et al., 2016; Shi et al., 2012). Unrepaired TOP2-DPCs block the progression of RNA and DNA polymerases. This can lead to accumulation of recombinogenic intermediates that can catalyze the generation of deletions and translocations. TOP2-DPCs can therefore lead to mutagenesis, neurological diseases, and carcinogenesis (Riccio et al., 2020).

Previously we identified seven point mutations in the *C. elegans* ortholog of TDP2 ( *tdpt-1)* that were responsible for suppression of *top-2(it7)* induced male-specific meiotic chromosome segregation defects (Bhandari et al., 2020). Interestingly, *tdpt-1* has not been previously reported to be involved in meiosis. To investigate the localization and expression of TDPT-1, we had a fluorescent protein and a polypeptide tag inserted at different termini of the TDPT-1 protein via CRISPR/Cas9 genome editing and assessed their functionality for further use in worms. The embryonic viability assays showed that CRISPR/Cas9 genome edited worms harboring either of the tagged forms, N-terminally tagged (wrmScarlet::TDPT-1) and C-terminally tagged (TDPT-1::3XFLAG), did not affect survival of progeny compared to wild type (N2) [N2: 95.2%, *wrmScarlet::tdpt-1* : 95.5%, and *tdpt-1::3xflag* : 96%; p=0.46 for N2 vs. *wrmScarlet::tdpt-1* and p=0.25 for N2 vs. *tdpt-1::3xflag* , Figure 1D]. However, *tdpt-1* is not an essential gene. Our previous work showed that knockdown of *tdpt-1* via RNAi does not cause embryonic lethality and a phenotype associated with *tdpt-1* mutations is only revealed in the context of *top-2* mutations due to its DNA repair function (Bhandari et al., 2020). Future studies will analyze the function of the tagged TDPT-1 proteins in the presence of *top-2* mutations in both hermaphrodite and male meiosis. Live imaging of worms expressing wrmScarlet::TDPT-1 showed that TDPT-1 is expressed in both the soma and germ line. TDPT-1 was found within nuclei throughout the germ line and within the spermatheca (Figure 1A), somatic cell nuclei (Figure 1B), and in the nuclei of developing embryos (Figure 1C) in hermaphrodites at 20°C. As wrmScarlet::TDPT-1 exhibits the expected localization pattern (within nuclei for DNA repair functions), the addition of the wrmScarlet tag does not appear to disrupt the localization of the TDPT-1 protein.

To determine the utility of using the *tdpt-1::3xflag* strain for biochemical studies (an antibody against *C. elegans* TDPT-1 protein is not available), we performed Western blots on whole worm lysates from wild-type (N2) and *tdpt-1::3xflag* hermaphrodites at 20°C and probed with anti-FLAG antibodies. A ~44 kDa band was identified in the lysates from *tdpt-1::3xflag* worms that was not present in wild-type lysates ( *C. elegans* TDPT-1 is 40.9 kDa (Rao et al., 2016), Figure 1E). In conclusion, our data show that TDPT-1 tagged strains can be used to investigate the localization of TDPT-1 protein in live animals, as well as to evaluate endogenous TDPT-1 protein levels, and can therefore be utilized in further experiments.

## Methods

**CRISPR/Cas9 generation of endogenously tagged TDPT-1 strains**

C-terminally tagged wrmScarlet::TDPT-1 (PHX2976) and N-terminally tagged TDPT-1::3XFLAG (PHX2925) were generated via CRISPR/Cas9 genome editing technology by SunyBiotech Corporation (Fu Jian Province, China).

**Live imaging**

Spinning disk confocal live imaging was performed by placing 6-8 worms on a slide containing a freshly grooved 2% agarose pad prepared using a vinyl record (Rivera Gomez and Schvarzstein, 2018) and 5 µl of anesthetic (2mM tetramisole in M9 buffer). Adult hermaphrodites were imaged on an AndorDragonfly inverted spinning disk confocal microscope using 40x/1.3 oil objective. Differential interference contrast (DIC) and 561 nm laser directed Z-stack images were captured using an Andor Zyla 4.2 sCMOS camera. Image analysis and editing was conducted using Fiji Is Just ImageJ (Schindelin et al., 2012) and Photoshop.

**Embryonic viability assay**

For the embryonic viability assay, individual N2, *wrmScarlet::tdpt-1* , and *tdpt-1::3xflag* L4 hermaphrodites were picked to single 30 mm MYOB plates spotted with *E. coli* OP50 and incubated at 20°C to allow embryo laying. Each hermaphrodite was then transferred to a new individual plate every 24 h until all the embryos were laid. All plates were counted for larvae and unhatched embryos 24 h post adult transfer. Embryonic viability was determined as the number of larvae divided by the total number of embryos laid. Three independent experiments were performed for each strain.

**Western Blotting**

20 L4 worms from each strain were allowed to grow on ten 100 mm plates at 20°C until the plates were covered with worms. After washing with M9 buffer, the worm pellets were flash frozen and stored at -80°C. Protein extraction was performed through sequential processes of pellet grinding, resuspension in lysis buffer, sonication and centrifugation as described (Zanin et al., 2011). 15 µL of each sample was loaded onto a 15% cast polyacrylamide gel (Bio-Rad Laboratories, Hercules, CA), run at 200V, transferred onto a 0.45 µm nitrocellulose membrane (Bio-Rad Laboratories, Hercules, CA) and blocked using 5% milk (in TBST) for 1 h. The membranes were blotted for TDPT-1::3XFLAG using mouse anti-FLAG (Sigma-Aldrich, #F1804-200UG) primary antibody (1:500 dilution in 5% milk/TBST) and for anti-α Tubulin (DM1α Sigma-Aldrich, #T9026) primary antibody (1:10,000 dilution in milk/TBST) overnight at 4°C. Membranes were then washed with TBST, incubated in secondary antibodies (anti-mouse HRP-conjugated antibodies, Life Technologies, 1:10,000 dilution in TBST), at room temperature for 2 h. Finally, blots were exposed to Clarity MAX ECL Western blotting substrate and were imaged with a ChemiDoc Imaging System (Bio-Rad Laboratories, Hercules, CA).

## Reagents

N2

PHX2976 *tdpt-1(syb2976) [wrmScarlet::tdpt-1]*

PHX2925 *tdpt-1(syb2925) [tdpt-1::3xflag]*

N2 is available from the CGC, TDPT-1 strains are available from the Jaramillo-Lambert Lab.
